# Influenza Virus Infection Induces Host Pyruvate Kinase M Which Interacts with Viral RNA-Dependent RNA Polymerase

**DOI:** 10.3389/fmicb.2017.00162

**Published:** 2017-02-09

**Authors:** Yukari Miyake, Kosuke Ishii, Ayae Honda

**Affiliations:** Department of Frontier Bioscience, Hosei UniversityTokyo, Japan

**Keywords:** influenza virus, RdRp, pyruvate kinase M, RNA synthesis, PA subunit, yeast two-hybrid screening, PKM2, PKM1

## Abstract

Influenza virus RNA-dependent RNA polymerase (RdRp) is a heterotrimer of three viral proteins, PB1, PB2, and PA and is involved in both transcription and replication of the negative strand of the viral RNA (vRNA) genome. RdRp is multifunctional, possessing RNA polymerase, cap binding, and endonuclease activities. The enzyme synthesizes three different RNAs, complementary RNA (cRNA) and messenger RNA (mRNA) from vRNA, and vRNA from cRNA. To synthesize these three RNAs, RdRp requires conversion of its function by host factor. Here, we performed yeast two-hybrid screening to identify the relevant host factor, revealing that pyruvate kinase M2 (PKM2) interacted with the PA subunit of influenza virus RdRp. PKM2 is one of two enzymes (PKM1 and PKM2) produced by alternative splicing of the pyruvate kinase M (*PKM*) pre-mRNA. We determined the interacting regions in both PKM2 and PA, the expression level of PKM by western blotting at different time points after viral infection, and the effects of transfection of siRNA targeting PKM on influenza virus replication. The results demonstrated that the C-terminal region of PKM2 interacted with the C-terminus of the PA subunit, that the expression level of PKM2 increased with influenza virus infection time, and that this enzyme is essential for influenza virus multiplication. Moreover, isoelectric focusing of uninfected and influenza virus infected cell extracts, followed by gradient gel electrophoresis to separate the PKM1 and PKM2 isoforms and western blotting indicated that PKM2 became more acidic after influenza infection. The decreased pH of PKM2 may have been due to phosphorylation, and phosphorylated PKM2 is active as a pyruvate kinase and protein kinase; therefore, it is possible that PKM2 may transfer a phosphate group to PA and consequently transform the function of RdRp from transcriptase to replicase.

## Introduction

The genome of influenza virus is composed of eight negative RNA molecule segments (Palese and Shaw, 2007). RNA-dependent RNA polymerase (RdRp) binds to the promoter region of each segment of genome RNA and nucleoprotein (NP) binds along the genomic RNA (Honda and Ishihama, 1987/1997). The influenza virus invades the cytoplasm of host cells in endosomes by binding to sialic acid on cellular membranes via hemagglutinin (HA), a viral glycoprotein on the envelope of the influenza viral particle (Lakadamyali et al., 2004), and is transported to the vicinity of the nucleus, where decapsidation of the virus occurs and a vRNP complex, consisting of viral RNA (vRNA), RdRp, and NP is released from the endosome (Lakadamyali et al., 2004). Replication and transcription of vRNA occur in the nucleus. RdRp catalyzes the synthesis of three species of RNA (Krug et al., 1989): complementary RNA (cRNA), messenger RNA (mRNA), and vRNA. Initiation of mRNA synthesis requires host capped RNA as a primer, while neither vRNA nor cRNA synthesis rely on a primer for initiation. In viral particles, RdRp synthesizes mRNA but not cRNA or vRNA (Honda et al., 1990); while in virus-infected cells, the same enzyme synthesizes not only mRNA, but also cRNA and vRNA.

RNA-dependent RNA polymerase is a heterotrimer composed of the viral proteins PB1, PB2, and PA. PB1 plays a key role in both RdRp assembly and the catalytic function of RNA synthesis, PB2 interacts with the cap structure of host cell mRNA (Krug et al., 1989; Honda et al., 1999), and PA has both endonuclease activity when bound with PB2, and genomic RNA promoter binding activity when bound with PB1 (Dias et al., 2009). Purified RdRp from *Trichoplusia ni* Tn5 cells coinfected with recombinant baculoviruses expressing the three RdRp subunits (PB1, PB2, and PA) could synthesize only mRNA, but not the two other RNA species (Honda et al., 2002). RdRp generated from its three subunits encoded by an expression vector transfected into cells can synthesis all of the three different RNA species (Nakagawa et al., 1996). These results indicate that RdRp must be converted by some host factor(s) to enable catalysis of the synthesis of the three types of RNA. Therefore, we performed yeast two-hybrid screening to identify the host factor involved in the conversion of RdRp. Our results identified several candidate proteins, among which the host protein, PKM2, was found to interact with the C-terminus of the PA subunit, which interacts with the PB1 subunit (Toyoda et al., 1996).

Pyruvate kinase (EC 2.7.1.40) catalyzes the formation of pyruvate and ATP from phosphoenolpyruvate (PEP) and ADP, and is an important enzyme in the regulation of glycolysis (Llorente et al., 1970). The pyruvate kinase M (*PKM*) gene consists of 12 exons. The pyruvate kinase M1 (*PKM1*) and pyruvate kinase M2 (*PKM2*) isoforms are produced by exclusive alternative splicing of PKM pre-mRNA (Noguchi et al., 1986) and they are both composed of 11 exons, lacking exons 10 and 9, respectively, with a molecular size difference between the encoded proteins of approximately 1.0 kDa. In the nucleus, PKM2 regulates cell proliferation (Hoshino et al., 2007) and binds directly to phosphorylated histone H3, thus promoting gene transcription (Yang et al., 2012).

The present study was performed to identify the host factor that interacts with the PA subunit of RdRp, and to determine its expression level and modification during influenza virus infection. Our results indicated that PKM protein expression was induced by influenza virus multiplication, and that PKM was required for virus multiplication. We also determined that the C-terminal region of PKM2 interacted with the C-terminal region of the PA subunit. Following influenza virus infection, the pH of PKM1 became basic, while that of PKM2 became more acidic, indicating that the interaction between PKM2 and PA may result in transfer of a phosphate group from PKM2 to the PA subunit of RdRp and the consequent conversion of the function of RdRp from transcriptase to replicase.

## Materials and Methods

### Yeast Two-Hybrid Screening

As an initial screen for host proteins that interact with RdRp, the full-length cDNAs of each influenza virus RNA polymerase subunit were inserted into the bait vector, pHybLex/Zeo, for expression as fusion proteins with the LexA DNA-binding domain, while a HeLa cell cDNA library (Invitrogen, USA) was inserted into the prey vector, pYes/Trp2, to construct an expression plasmid library encoding proteins fused with a transcription activation domain. After co-transfection of the two types of plasmid into *Saccharomyces cerevisiae* L40, transformants were subjected to first screening on selection medium containing 300 mg/mL Zeocin and without tryptophan, histidine, or lysine. Viable colonies were picked and subjected to second round of screening for detection of β-galactosidase activity. Zeocin-resistant and β-galactosidase-positive cDNA clones were isolated. For confirmation, two-hybrid screening was performed using the opposite combinations (i.e., positive clones fused to the DNA binding domain and RdRp domains fused to the activation domain). Finally, cDNAs of positive clones were sequenced.

Yeast two-hybrid screening was also performed to confirm the interactions between the identified HeLa cell proteins and influenza virus RNA polymerase, and to map the sites of contact sites between the two proteins. For these experiments, cDNAs encoding the influenza virus RNA polymerase PA subunit and segments thereof were inserted into the pHybLex/Zeo vector for expression as respective DNA-binding domain fusion proteins, while full length and partial *PKM2* cDNAs were expressed as fusion proteins with the activation domain.

### Cells and Virus

H292 cells (human lung epithelial cell, purchased from ATCC) were maintained in Eagle’s Minimum Essential Medium (EMEM; Nissui, Japan) with 10% Fetal Bovine Serum (FBS, Gibco, USA) in a humid incubator at 37°C and 5% CO_2_ (Thermo, USA). Influenza virus A/PR/8/34 was used throughout the study.

### Virus Infection

Semi-confluent H292 cells were infected with influenza virus at multiplicity of infection (MOI) of 1, followed by washing with EMEM (pH 7) without FBS. Virus adhesion was carried out in a humid incubator at 34°C and 5% CO_2_ for 1 h, corresponding to 0 h post-infection (hpi). After incubation for 1 h, the viral solution was removed and replaced with EMEM containing 10% FBS and incubation continued for the required time.

### Western Blotting Assay

Uninfected and virus infected cells were removed from culture vessels using 0.05% trypsin/0.04% EDTA/PBS and washed with PBS three times by centrifugation at 100 g (Tomy MX301, Japan) at 4°C for 5 min. The cell pellet was disrupted in 100 μL hypotonic buffer containing 20 mM HEPES (pH 7.5), 1.5 mM MgCl_2_, 0.1% Triton X-100, and 10 mM KCl with glass milk in Precelly 24 (Bertin, France) at 6500 rpm for 20 s, and then placed on ice for 5 s; this combined step was repeated seven times. The disrupted cell pellet was centrifuged at 21130 g for 30 min at 4°C. The resultant supernatant was transferred into new 1.5 mL tubes.

To investigate PKM expression in uninfected and influenza infected cells, and in siRNA experiments, aliquots of supernatant corresponding to 10^5^ cells were separated by 8% sodium dodecyl sulfate-polyacrylamide gel electrophoresis (SDS-PAGE) followed by blotting on to polyvinylidene fluoride (PVDF) membrane (Nippon Genetics, Japan) with 5 mM N-cyclohexyl-3-aminopropanesulfonic acid (CAPS; Dojindo, Japan) buffer at 4°C and 0.25 A for 1 h. The blotted membranes were blocked with 5% skim milk/PBS for 1 h, washed, and then incubated in 1000x diluted rabbit anti-PKM antibody (Abcam, USA) for 1 h at 37°C. The membranes were then incubated with horseradish peroxidase-conjugated anti-rabbit IgG for 1 h at 37°C, followed by detection with chemiluminescence using an LAS 3000 mini imaging system (Fuji Film, Japan). The relative amount of PKM was calculated by the intensity of each band measured using the LAS 3000 mini software.

For separation of the PKM1 and PKM2 isoforms following isoelectric, proteins were separated by SDS-PAGE, using 6–15% gradient gels at 30 mA for 90 min. After SDS-PAGE, the proteins in the gel were blotted onto PVDF membranes at 0.2 A, 10°C for 1 h, and reacted with anti-PKM antibody (Abcam, USA) and then horseradish peroxidase-conjugated anti-mouse IgG followed by chemiluminescence detection using an LAS 3000 mini imaging system.

### Separation of Cytoplasmic Proteins by Isoelectric Focusing

Prior to separation of cytoplasmic proteins by isoelectric focusing, influenza virus-infected and uninfected cells were harvested by trypsinization and centrifugation at 100 g, and the pellets disrupted in 5 M urea solution with glass milk in Precelly 24, as described in section 2.4. The resultant supernatant was further centrifuged at 186000 g (Beckman, Optima ultracentrifuge, USA) for 1 h at 4°C. Aliquots of 1 mg of supernatant protein were applied to equilibrated Bio-Lyte (pH 3–10; Bio-Rad, USA). Protein separation was performed in a Rotofor (Bio-Rad, USA). A pre-run was performed at 3 W using 20 mL of MilliQ water and isoelectric focusing was performed at 8 W for 4–6 h/15 W, 4°C, 60 min/8 W and then 10°C for 30 min. After separation, 20 samples were fractionated into 5 mL tubes. The pH of each sample was measured using a pH meter (Horiba, Japan) and fractions were assayed by western blotting (see Western Blotting Assay). We performed this experiment three times to confirm the results.

### siRNA Transfection

H292 cells in exponential growth phase were treated with 0.05% trypsin for 30 s followed by removal of excess trypsin and further incubation for 15 min at 37°C, harvesting by centrifugation at 100 g, and resuspension in buffer R, which was supplied with a commercial kit (Neon^®^ Transfection System, Thermo Fisher, USA). Aliquots of 5 × 10^5^ cells were mixed with 5 nmol of siRNA targeting *PKM2* and transfected using a Microporator (Digital Bio, Korea) at 1600 V, 20 ms, once. After siRNA transfection, the electroporated cells were resuspended in EMEM supplemented with 10% serum and cultured for 24 h. Cells were then infected with influenza virus at an MOI of 1. The assay was repeated three times. Cell lysates were subject to SDS-PAGE and western blotting to detect PKM (see Western Blotting Assay).

## Results

### The Influenza Virus RdRp PA Subunit Interacts with Pyruvate Kinase M

To understand the mechanism underlying the conversion of influenza virus RdRp function in host cells, we carried out yeast two-hybrid screening to identify host factor(s) interacting with RdRp and which RdRp subunit(s) were involved in the interaction using recombinant plasmids (Honda et al., 2007), as described in Section “Yeast Two-Hybrid Screening.” Screening of positive (interacting) yeast colonies demonstrated that the full-length PA expression vector interacted non-specifically with all vectors. Therefore, as shown in previous paper (Honda et al., 2007) we prepared cDNA clones encoding four fragments of PA (**Table 1**) in pHybLex/Zeo, and used these to screen for interacting host factor(s). Nine positive clones were obtained after screening, one of which contained the cDNA, *PKM2*. We tested whether PB1 or PB2 interacted with PKM. As shown in **Table 1**, both PB1 and PB2 did not interact with PKM.

**Table 1:** Identification of RNA-dependent RNA polymerase (RdRp) subunits that interact with pyruvate kinase M (PKM).

Influenza virus RdRp subunits (bait)	PKM (prey)
PB1	-
PB2	-
PA (1–346)	-
PA (347–452)	-
PA (453–638)	-
PA (639–716)	+

*Numbers indicate amino acid number from the N-terminus. -, no β-Gal activity detected; +, β-Gal activity detected.*

**FIGURE 1:**
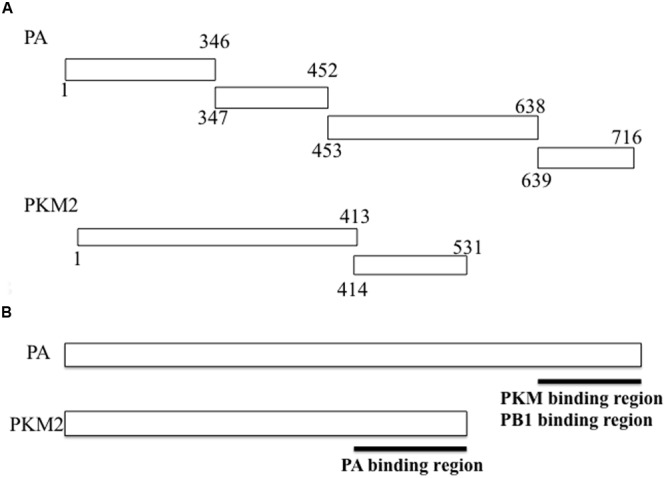
**Fragments of RNA-dependent RNA polymerase (RdRp) PA subunit and pyruvate kinase M (PKM) cloned into vectors for use in yeast two-hybrid screening assays. (A)** For confirmation of the PA-PKM2 interaction and mapping of the contact site on each protein, cDNA sequences encoding each of the indicated PA regions were inserted into the pHybLex/Zeo “bait” plasmid for expression as fusion proteins with the DNA-binding domain, while cDNA sequences encoding each PKM2 fragment were inserted into the pYESTrp2 “prey” plasmid for expression of fusion proteins with the activation domain. After co-transfection of bait and prey plasmids into *Saccharomyces cerevisiae* L40, a β-galactosidase assay was carried out. Results are summarized in **Tables 1** and **2**. **(B)** The interacting regions between PA and PKM2 were mapped.

The region of PKM2 that interacted with the PA subunit was then identified by a further yeast two-hybrid screening experiment. As shown in **Table 2**, the C-terminal region of PA interacted with the C-terminal region of PKM2. In addition, we also constructed two pHybLex/Zeo vectors carrying inserts corresponding to N- and C-terminal regions of PKM2 (**Figure 1A**), and examined the interaction of these domains with the C-terminal region of PA. The results indicated that C-terminal region of PA interacted with the C-terminus of PKM2 (**Figure 1B**).

**Table 2:** Identification of interaction sites between PA and PKM.

PA fragment (bait)	PKM (1–413; prey)	PKM (414–531; prey)
PA (1–346)	-	-
PA (347–452)	-	-
PA (453–638)	-	-
PA (639–716)	-	+

*Numbers indicate amino acid number from the N-terminus. -, no β-Gal activity detected; +, β-Gal activity detected.*

### Multiplication of Influenza Virus is Induced by PKM

**FIGURE 2:**
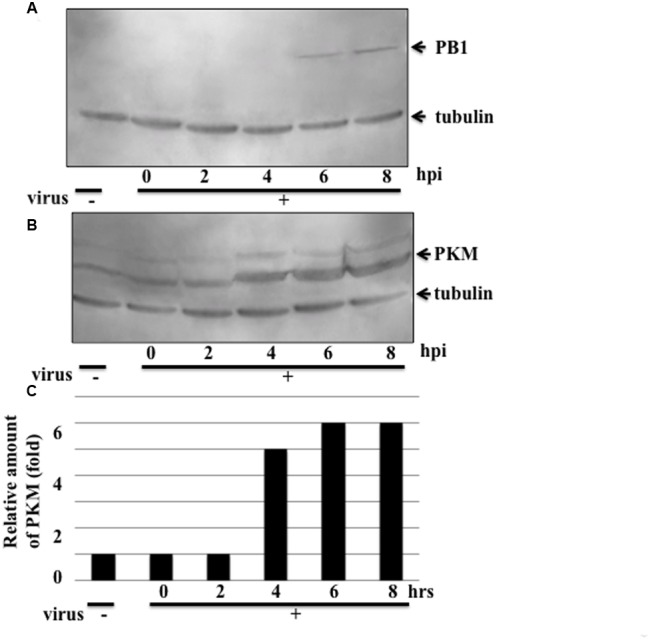
**Expression levels of the influenza virus RdRp PB1 subunit and PKM.** Influenza virus-infected cells were harvested at various time points and proteins separated by 8% SDS-PAGE, blotted onto PVDF membranes, and then reacted with anti-PB1 and anti-PKM antibodies, followed by chemiluminescence detection. 0, 2, 4, 6, and 8 hpi indicate time after influenza virus infection. Tubulin was detected as an internal marker. **(A)** PB1 expression **(B)** PKM expression **(C)** Relative amount of PKM, calculated from band intensities, standardized by the intensity of tubulin.

Next, we performed western blotting to investigate whether influenza virus multiplication induced PKM2 expression. H292 cells were infected with influenza virus at an MOI of 1 and harvested at different time points (0, 2, 4, 6, and 8 hpi). Western blotting of cell lysates with anti-PKM antibody indicated that the PKM expression level in influenza virus-infected cells was increased by more than fivefold compared to uninfected cells at 4 hpi; however, expression of the viral PB1 subunit was detected later than that of PKM (at 6 hpi) (**Figure 2**). These observations indicate that PKM expression was induced at an early stage of viral multiplication. The timing of PKM expression time after virus infection was similar to that of Ebp1, ErbB3 (an epidermal receptor tyrosine kinase)-binding protein and also interacted with PB1 subunit of RdRp (Ejima et al., 2011), and the elevated levels of PKM lasted until 8 hpi.

### PKM was Modified by Influenza Virus Multiplication

**FIGURE 3:**
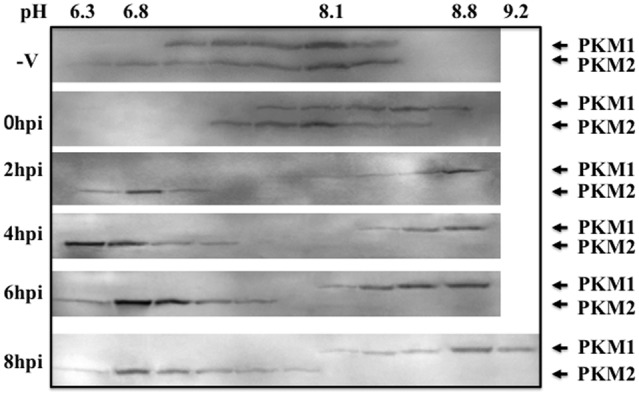
**Isoelectric focusing analysis of PKM.** Cells were disrupted at various time points following influenza virus infection (0–8 hpi) and the proteins separated in a Rotofor by isoelectric focusing, using an ampholyte (pH 3–10). After separation by isoelectric focusing, solutions were fractionated into 20 tubes. The pH of each tube was measured using a pH meter (Horiba). Next, 40 μL of each fraction was separated by SDS-PAGE on 6–15% gradient gels to separate the two PKM isoforms (PKM1 and PKM2), followed by blotting and immunostaining using anti-PKM antibody with chemiluminescence detection.

To examine modification of PKM during influenza virus multiplication, the cytoplasmic proteins isolated at various times after infection were separated in isoelectric focusing solution using a Rotofor. The molecular masses of the PKM1 and PKM2 isoforms are approximately 58 and 57 kDa, respectively. To visualize the size difference, the proteins were separated by SDS-PAGE on 6–15% gradient gels. Western blots of the separated protein fractions probed with anti-PKM indicated that the pH values of both PKM1 and PKM2 obtained from uninfected cells were close to 8.1 (**Figure 3**). However, during adhesion of influenza virus particles for 1 h at 34°C (at the 0 hpi timepoint), PKM1 became more basic, while PKM2 remained at pH 8. Interestingly, PKM2 shifted to the acidic region at 2 hpi, while PKM1 shifted further into the basic region (pH 9) (**Figure 3**).

### Influence of PKM on Influenza Virus Replication

**FIGURE 4:**
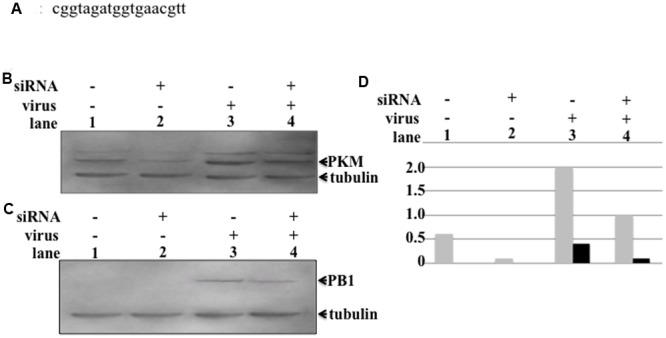
**Influence of PKM on influenza virus multiplication.** PKM siRNA was transfected into semi-confluent cells by electroporation, followed by incubation overnight in a humid, 5% CO_2_ incubator at 37°C. After overnight incubation, influenza virus-infected cells were transfected with siRNA at an MOI of 1. At various time points after infection, cells were harvested and disrupted, and proteins separated by 8% SDS-PAGE, followed by blotting onto PVDF membranes. The membranes were then reacted with anti-PB1 and anti-PKM antibodies, followed by chemiluminescence detection. **(A)** The sequence of the siRNA targeting PKM2. **(B)** Lanes 1 and 2, PKM expression level in uninfected cells (lane 1, siRNA-untransfected cells and lane 2, siRNA-transfected cells). Lanes 3 and 4, PKM expression level of virus-infected cells (lane 3, siRNA-untransfected cells; lane 4, siRNA-transfected cells). **(C)** PB1 expression levels of uninfected and virus-infected cells. Lanes 1 and 2, PB1 expression level in virus uninfected cells (lane 1, siRNA-untransfected cells; lane 2, siRNA-transfected cells). Lanes 3 and 4, PB1 expression levels of virus-infected cells (lane 3, siRNA-untransfected cells; lane 4, siRNA-transfected cells). **(D)** Relative amount of PKM and PB1, calculated from band intensities, standardized by the intensity of tubulin; gray bar represents PKM, black bar represents PB1.

Influenza virus replication consumes large amounts of nucleic acids for synthesis of vRNA, cRNA, and mRNA to produce progeny virus. The process of synthesizing large quantities of RNA also requires huge amounts of energy, for which ATP production is necessary, which requires either high levels or activation of PKM. To assess the pyruvate kinase level required for influenza virus multiplication, we designed an siRNA targeting PKM (**Figure 4A**). To reduce the levels of PKM in host cells, 5 nmol of siRNA was transfected into H292 cells as described in Section “siRNA Transfection.” The PKM levels in siRNA-transfected cells were decreased by more than fourfold compared to untransfected cells (**Figures 4B,D**, lanes 1 and 2, respectively). siRNA-transfected H292 cells were infected with influenza virus at an MOI of 1, and at 6 hpi, the influenza virus-infected cells were harvested, and PB1 expression examined by western blotting. As shown in **Figures 4C,D**, lanes 3 and 4, the expression levels of PB1 were lower in siRNA-transfected, compared with untransfected, cells. These results indicate that PKM is important for influenza virus replication.

## Discussion

The RdRp enzyme can catalyze the synthesis of three species of RNA (Krug et al., 1989); however, purified RdRp from recombinant baculovirus coinfected with Tn5 requires a primer for RNA synthesis, and cannot synthesize all three different RNAs. In contrast, purified heterotrimer from recombinant baculovirus coinfected with Tn5 exhibited both primer-dependent and independent RNA synthesis (Honda et al., 2002), indicating that influenza virus RdRp requires some host factor(s) to synthesize the three species of RNA.

From yeast two-hybrid screening, the host protein, PKM2, was shown to interact with the PA subunit of RdRp. PKM2 catalyzes transfer of a phosphate group from PEP to ADP, yielding one molecule of pyruvate and one molecule of ATP (Altenberg and Greulich, 2004). Investigation of PKM protein levels indicated that its expression was induced by influenza virus infection at 4 hpi before viral protein detection by western blotting (**Figures 2A,B**). PKM is an important enzyme for generation of ATP, and influenza virus replication involves the synthesis of large amounts of vRNA, cRNA, and mRNA, which requires copious quantities of ATP; therefore, the induction of PKM expression by influenza virus infection is logical. At 4 hpi, the expression of PKM was increased and influenza virus transcription and replication also increased at this time point, hence these two phenomena were correlated. This result indicates the possibility of phosphorylation of the PA subunit by PKM; however, our results do not determine whether PKM2 interacts with the PA subunit as a monomer or as part of the RdRp complex, to alter the function of RdRp. To answer this question our future experiments will include phosphorylation assays of the PA subunit along with assays to determine whether the function of RdRp can be altered by interaction with PKM2.

York et al. (2014) reported that serine/threonine protein phosphatase 6 (PP6) interacts with PB1 and PB2 subunits directly and demonstrated that cells in which PP6 was knocked-down using siRNA exhibited reduced vRNA accumulation and attenuated viral growth. Their results indicate that phosphate has an important role in influenza virus replication. Our result is the first report of the interaction between PKM2 and the PA subunit, and of reduction of PKM2 expression resulting in decreased influenza virus protein expression.

Pyruvate kinase M2 phosphorylates histone H3 and promotes gene transcription and tumorigenesis (Yang et al., 2012). The PKM2 dimer functions as a protein kinase and promotes cell proliferation (Gao et al., 2012). As shown in **Figure 3**, following influenza virus infection, PKM1 became more basic, while PKM2 became more acidic. The decreased pH of PKM2 may have been due to phosphorylation, and phosphorylated PKM2 is active as a pyruvate kinase and protein kinase. The protein kinase function of PKM2 may be important for influenza virus replication. Knock-down of the *PKM2* gene decreased EGFR phosphorylation (Hsu et al., 2016) and PKM2 can translocate into the nucleus and interact with the HIF-1α subunit to transactivate target genes (Dong et al., 2014). These functions of PKM2 suggest a very attractive hypothesis that PA in RdRp may be phosphorylated by PKM2, leading to the conversion of the function of RdRp from transcriptase to replicase, as phosphorylated PKM2 may transfer a phosphate group to PA.

In our previous report, we determined that the PA-PB1 heterodimer functions as a replicase. Based on this result, we speculated that the PA subunit may have an important role in the replicase function of RdRp. In this study, we did not directly demonstrate PA subunit modification by interaction with PKM2; however, the PA subunit of RdRp may be phosphorylated and function as a converter of RdRp from a transcriptase to a replicase. We are currently engaged in experiments to clarify whether the function RdRp can be converted by phosphorylation of PA by PKM2. In support of this hypothesis, depletion of pyruvate kinase using siRNA targeting PKM2 inhibited influenza virus replication (**Figures 4B,C**). It will be necessary to assess the mechanism underlying the inhibition of virus replication by depletion of PKM. Further studies are required to determine whether phosphorylated PKM2 transfers a phosphate group to PA and induces an alteration in RdRp function.

## Author Contributions

YM carried out western blotting. KI performed protein extraction from cells and 2D assay. AH designed this experiment and wrote the manuscript.

## Conflict of Interest Statement

The authors declare that the research was conducted in the absence of any commercial or financial relationships that could be construed as a potential conflict of interest.
